# Effect of the Electrodeposition Potential on the Chemical Composition, Structure and Magnetic Properties of FeCo and FeNi Nanowires

**DOI:** 10.3390/ma18112629

**Published:** 2025-06-04

**Authors:** Anna Nykiel, Alain Walcarius, Malgorzata Kac

**Affiliations:** 1 Institute of Nuclear Physics, Polish Academy of Science, PL-31342 Kraków, Poland; anna.nykiel@ifj.edu.pl; 2 Université de Lorraine, CNRS, Laboratoire de Chimie Physique et Microbiologie pour les Matériaux et l’Environnement (LCPME), F-54000 Nancy, France; alain.walcarius@univ-lorraine.fr

**Keywords:** FeCo, FeNi, nanowires, template-assisted electrodeposition, magnetic properties

## Abstract

This study focused on investigations of FeCo and FeNi nanowires prepared by template-assisted electrodeposition in polycarbonate membranes. Nanowires with a diameter of 100 nm and length of 6 µm were grown at different cathodic potentials and electrolyte compositions. Scanning electron microscopy images revealed densely packed arrays of continuous nanowires with smooth surfaces without visible porosity, regardless of the applied potential. Chemical analysis of nanowires pointed out weak sensitivity of chemical composition on the electrodeposition potential in the case of FeCo nanowires, in contrast to FeNi nanowires, where the increase of the cathodic potential resulted in higher Ni content. X-ray diffraction studies showed polycrystalline structure for all samples indicating B2 phase (Pm-3m) with isotropic growth of FeCo nanowires and FeNi_3_ phase with a preferential growth along [111] direction in the case of FeNi nanowires. The peak broadening suggests a fine crystalline structure for both FeCo and FeNi materials with average crystallite sizes below 20 nm. Magnetic studies indicated an easy axis of magnetization parallel to the nanowire axis for all FeCo nanowires and potential-dependent anisotropy for FeNi nanowires. The present studies thus suggested the feasibility of producing segmented nanowires based on FeNi alloys, while poor chemical sensitivity to the applied potential was observed for the FeCo system.

## 1. Introduction

Nanowires (NWs) are one-dimensional objects that have different properties than their macroscopic counterparts, due to shape anisotropy and large surface-to-volume ratio. Particularly fascinating thanks to their magnetic, optical, and electrical properties, magnetic NWs find many applications from biomedicine to customer electronics [1]. The main parameters determining the use of NWs are their geometry and chemical composition, which strongly affect the physical properties of these nanostructures, especially regarding their magnetic behavior [2]. Such objects with appropriate remanence and coercivity can be used to create magnetic memory units with perpendicular anisotropy. Magnetic NWs also seem to be the most promising candidates for domain wall guide in a new generation of magnetic memory units called racetrack memories, in which domain walls are used to store, transmit and manipulate binary information [3,4,5,6,7]. To this end, the domain walls must be precisely located and anchored at selected positions to ensure thermal stability and avoid undesired domain wall drift [8,9]. These requirements can be met by a nanowire matrix composed of non-interacting segmented NWs.

A matrix of quasi-non-interacting NWs can be produced by the template-assisted electrodeposition technique. This is the most efficient and inexpensive method, enabling the production of ordered NWs with geometry defined by the template characteristics. Templates in the form of porous membranes of alumina [10], silica [11], or polycarbonate [12] produced by different chemical or physical processes are characterized by various surface pore density, aspect ratio, pore diameter, size distribution, and pore arrangement, which are attractive for NWs growth [13]. Among them, polycarbonate membranes seem to be an interesting template with relatively large distances between the pores, providing negligibly small inter-wire interactions as dipole-dipole interaction decreases with the cube of the distance [14]. Too small inter-pore spacing increases the magnetostatic interaction between nanowires, which significantly reduces the coercivity compared to the quasi-isolated nanowires and creates a risk of undesirable magnetic switching [15,16]. Moreover, polycarbonate is a biocompatible material widely used in biomedicine and daily applications without any toxicity or side effects in contact with the skin [17].

Segmented nanowires with well-defined segment boundaries that act as pinning sites can be produced from a single electrolyte bath, by applying different cathodic potentials [18,19]. For this purpose, an electrodeposition medium containing at least two kinds of metal ions with relatively distinct reduction potentials will be required [13]. Examples of such systems containing two magnetic elements are FeCo and FeNi alloys. The multi-component materials with various chemical compositions enable the production of nanowires with a relatively easy-to-control structure and magnetic properties [20,21]. Previous studies dealing with the effect of electrodeposition conditions on the chemical composition of such deposits have been reported for coatings [22] or nanowires produced in alumina membranes [23], but the influence of the applied potential is poorly investigated in the case of nanowires deposited in polycarbonate membranes.

In the concentration range investigated here (see experimental section), FeCo alloys are expected to crystallize in an ordered BCC (body-centered cubic) structure (B2) [24]. They exhibit a relatively low value of the magnetic anisotropy constant, which turns out to be much higher when the structure transforms to a body-centered tetragonal structure [25] under the influence of stress or impurity doping [26]. The increase in Co content results in a gain in the coercivity and squareness [27,28], but compared to pure Co, the alloyed nanowires show higher magnetization and lower coercivity, which extends their application range in biomedicine and consumer electronics [28,29,30,31].

The FeNi alloy structure, depending on the Ni content, changes from Ni solid solution in the Fe BCC structure to Fe solid solution in the Ni FCC (face-centered cubic) structure, the latter of which can evolve towards L1_2_ (cubic) or L1_0_ (tetragonal) ordered phases in specific concentration ranges. In the studied concentration range, two phases are predicted thermodynamically: (1) the structures of FeNi L1_0_ with Fe and Ni atoms occupying alternate position in the (002) planes (one layers of Fe atoms and next of Ni atoms) and (2) FeNi_3_ L1_2_ with Ni atoms located in the face-centered sites of the unit cell and Fe atoms in the corners. FeNi_3_ alloy known as permalloy is characterized by low coercivity and negligible magnetic anisotropy, but high magnetic permeability and saturation magnetization, significant remanence, magnetostriction close to zero, and relatively high Curie temperature (612 °C) [32,33,34,35]. Because of the differentiation of individual phase properties, the increase in the Ni content shows a complex behavior that depends on the Ni concentration range and the nanowire geometry [36,37].

The system geometry and nanowire structure also have a significant influence on the magnetic properties of the samples. Key parameters such as the aspect ratio and inter-wire distance determine the shape anisotropy and dipolar interactions between nanowires, which influence the easy axis direction, coercivity, and squareness [16,29,38,39,40,41,42]. To ensure the thermal stability of nanowires and optimal magnetic parameters, the array of nanowires in the diameter range of 50–100 nm should be considered [18]. Moreover, the elements being the components of the analyzed binary systems exhibit various crystallographic structures [43,44,45,46], characterized by different magnetocrystalline anisotropy constants, which in single crystalline or textured samples may significantly impact their magnetic behavior [47,48]. Magnetocrystalline anisotropy in competition with shape anisotropy and dipolar interactions affects the magnetic anisotropy of nanowires, which determines their application scope [27,43,44,49].

The idea for the production of 3D memory systems is based on the possibility of modifying the chemical composition of nanowires by tuning the applied cathodic potentials. However, changes in electrodeposition potentials affect the hydrogen evolution, which significantly increases at higher overpotentials, causing a more porous and defect-filled nanowire structure [13,50,51,52]. Such imperfections can lead to high coercivity due to the domain wall anchoring effect [14], but these additional pinning sites can suppress domain wall propagation in racetrack memory applications [53], requiring higher current densities for coherent domain wall motion [18]. On the other hand, Schöbitz et al. found that surface roughness, dislocations, and impurities are not expected to play a significant role in domain wall pinning [9]. Thus, the studies of nanowire morphology, their structure, and magnetic properties are crucial parameters that should be known in testing materials for potential application in racetrack memories.

Therefore, in this study, we will investigate the effect of electrodeposition conditions for the generation of FeCo and FeNi nanowires in polycarbonate membranes by analyzing the changes in their chemical composition and the related structural, morphological, and magnetic modifications caused by the application of various cathodic potentials. This will enable us to produce, from a single electrodeposition bath, nanowires composed of binary alloys with different elemental contents separated by a sharp boundary.

## 2. Materials and Methods

FeCo and FeNi nanowires were electrodeposited into commercially available polycarbonate (PC) membranes (Sterlitech Corporation, Kent, OH, USA) with a pore diameter of 100 nm (Φ) and a membrane thickness of 6 µm (L). The average inter-pore distance estimated at approximately 400–500 nm corresponds to a porosity of 3.1% (pore density of 6 × 10^8^ pores/cm^2^). The electrodeposition process was performed in a three-electrode system with Ag/AgCl and Pt as reference and counter electrodes. The working electrode was a membrane covered on one side with a sputtered Cu layer to close the pores and ensure electrical contact. The electrodeposition was monitored by an AUTOLAB PGSTAT302N (Metrohm Autolab B.V., Utrecht, The Netherlands) potentiostat operating in the potentiostatic mode.

The electrodeposition media consisted of aqueous solutions containing analytical grade chemicals at selected concentrations (as shown in Table 1) and were prepared based on deionized water (resistivity above 18 MΩcm) from the Millipore system (SAS-67120, Molsheim, France). The solution pH was adjusted using 2.5 M H_2_SO_4_ and 2 M NaOH and stabilized by boric acid. Additionally, ascorbic acid was added to prevent oxidation of Fe^2+^ species. The solution temperature during the electrodeposition process was kept at 20 °C. Nanowires were deposited at cathodic potentials ranging from −1 V to −2 V vs. Ag/AgCl (all potential values are given vs. Ag/AgCl). The electrodeposition process was continued until the membrane was completely filled, as evidenced by a sudden increase in the cathodic current.

After NW electrodeposition, the surfaces of the membranes were imaged using a Tescan Vega 3 (Tescan Orsay Holding, a.s., Brno, Czech Republic) scanning electron microscope (SEM with a detector of secondary electrons (to control the appearance of eventual overdeposition caps). The NW morphology, also examined by SEM, was observed after membrane dissolution in dichloromethane. An energy dispersive spectrometer (EDS) with a silicon drift detector (SDD) XFlash Detector 610M was used to study the chemical composition of nanowires (Bruker Nano GmBH, Berlin, Germany). Each analysis is an average of 10 measurements with a standard deviation of ±1. The homogeneity of the samples was observed on the EDS maps.

The structure of NWs was studied using X-ray diffraction (XRD) performed in θ–2θ geometry using a Philips X’Pert MRD Pro diffractometer (Malvern Panalytical Ltd, Malvern, UK) with Cu Kα radiation operating at 40 kV and 30 mA. In this investigation, we measured as-deposited samples with NWs embedded in the membrane coated on one side with a Cu layer. Based on the broadening of peaks in diffraction patterns, the crystalline size was determined using the Scherrer equation.

The magnetic properties of NWs were studied using a superconducting quantum interference device (SQUID) by Quantum Design, an MPMS magnetometer (San Diego, CA, USA) at an external magnetic field of up to 40 kOe applied in the membrane plane (H ꓕ NWs) and out of the membrane plane (H II NWs). The nanowires were analyzed at a temperature of 300 K and were kept in membranes during measurements. The diamagnetic signal from the sample holder and the polycarbonate was subtracted from the hysteresis loops. The magnetic moment was determined with an error of less than 2%, which is smaller than the symbol size in the figures.

## 3. Results and Discussion

FeCo and FeNi NWs were deposited at various cathodic potentials and solution compositions, which influenced their electrochemical behavior, chemical composition, structure, and magnetic properties, as described in the following sections.

### 3.1. Electrochemical Studies

**Figure 1:**
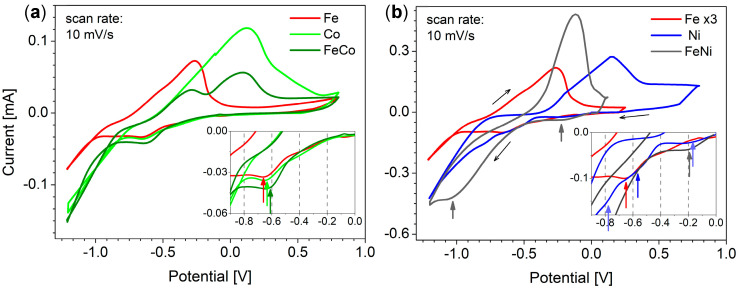
Cyclic voltammograms recorded in solutions containing FeSO_4_, CoSO_4_, and NiSO_4_ or their mixture in binary systems, with concentrations equal to 5 mM for each element in separate electrolytes, and 1 mM FeSO_4_, 3 mM CoSO_4_, and 5 mM NiSO_4_ for (**a**) Fe/Co and (**b**) Fe/Ni binary systems (the CV curve for Fe was magnified three times in part (**b**)). Insets show magnification of the cathodic regions of interest.

The cyclic voltammetry (CV) method was first used to understand the electrochemical behavior of the selected elements with the idea to help at optimizing the electrodeposition parameters. Figure 1 shows the current vs. potential curves (CV) performed using gold film electrodes immersed in various electrolytes containing FeSO_4_, CoSO_4_, NiSO_4_, or their mixtures (and boric acid as supporting electrolyte and ascorbic acid as anti-oxidant) with pH adjusted to approximately 3. The potential window ranged typically between −1.2 V and +0.8 V or even less for the FeNi system (−1.2 V and +0.2 V). In all measurements, the potential was scanned from positive to negative value, starting from 0 V, with a scan rate of 10 mV/s. The concentrations of separate FeSO_4_, CoSO_4_, and NiSO_4_ solutions were 5 mM each, while the media for binary systems were adjusted to keep a molar ratio of 1:3 for FeCo and 1:5 for FeNi.

CV measurements performed for separated species show clear cathodic peaks for Fe^2+^ and Co^2+^ reduction, respectively located at −0.66 V and −0.63 V, and wave for the reduction of Ni^2+^ starting at about −0.56 V (Figure 1a,b insets). As can be seen, these cathodic signals appear in the order consistent with standard potentials, i.e., first Ni, then Co, and Fe. The splitting of Ni^2+^ reduction signal into two waves is possibly due to two successive monoelectronic reduction steps [51,54], whereas the first weak pre-wave at less cathodic values (i.e., −0.2 V) can be due to the underpotential deposition of nickel on the gold surface [55]. With further potential increase towards more cathodic values (i.e., below −1.0 V), a sharp current increase indicates an intensive hydrogen evolution. On scan reversal, anodic stripping peaks are observed in the order opposite to the metal cation reduction, confirming that metallic deposits were indeed formed during the cathodic scan and electrochemically dissolved (oxidized) during the anodic scan. The rather low slope of anodic current increase suggests a rather slow dissolution process.

The described CV curves were compared with binary FeCo (Figure 1a) and FeNi (Figure 1b) systems. The CV curve obtained for the FeCo system seems to indicate a slightly easier reduction compared to the media with separate Fe^2+^ and Co^2+^ ions, with cathodic peak observed at potential of −0.61 V. This might suggest some stabilization of metal species in the alloy compared to separate Fe and Co elements. As expected, on scan reversal, one can observe two peaks for the FeCo alloy, corresponding to the anodic dissolution of each element.

In the case of the FeNi system, the reduction of metal ions also starts a bit earlier compared to the reduction of Ni^2+^ and Fe^2+^ in separate media, with a more intense pre-wave and all main reduction waves merging in a single one with significantly higher intensity, suggesting more efficient reduction when forming the alloy. The shape of the single anodic stripping peak visible on scan reversal suggests quite fast FeNi dissolution, and it surprisingly appeared at a position between the Fe and Ni anodic peaks (in separated electrolytes). This may result from the overlap due to larger deposits than for the FeCo system (compare current scale in part (a) and (b) of Figure 1) and faster FeNi stripping.

**Figure 2:**
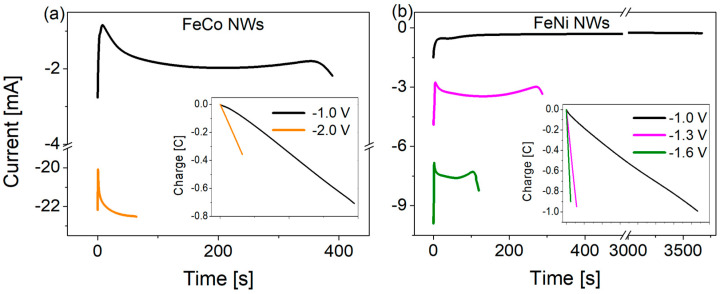
Variation of cathodic currents recorded as a function of time, as measured during the electrodeposition of (**a**) FeCo (higher ionic content) and (**b**) FeNi NWs with a diameter of 100 nm at different applied potentials. The insets show the corresponding electrical charge variations with time.

During the electrodeposition of FeCo and FeNi binary alloy nanowires in the polycarbonate membrane, the current response on the applied cathodic potential was measured as a function of time. Typical current vs. time plots for FeCo (a) and FeNi (b) NWs (Φ = 100 nm, L = 6 µm), generated from an electrodeposition medium with a higher content of Fe^2+^ and Co^2+^ ions (Table 1) and voltages ranging from −1.0 to −2.0 V, are presented in Figure 2. In both cases, the application of more cathodic potentials resulted in larger current increases and, consequently, shorter times needed to completely fill the pore channels. Nanowires prepared from solutions containing lower Fe^2+^ and Co^2+^ ion concentrations with the same molar ratio exhibited similar transients with significantly lower current values (due to the lower ion number) and jagged curves in case of a more negative voltage (due to ion deficiency). The corresponding electrical charge variations (insets in Figure 2) are linear, suggesting uniform growth of NWs inside the pores. The final charge values, i.e., the charge achieved after complete pore filling, decreased with increasing potential, which may indicate a more porous nanowire morphology and a lower degree of membrane filling due to the greater hydrogen evolution [13,56,57,58]. The same tendency was observed at lower Fe^2+^ and Co^2+^ ion concentrations.

The different values of the standard potential of individual redox couples result in various overpotentials and current densities for the reduction of metal ions, which are responsible for different deposition rates. A larger overpotential should cause the higher content of atoms with a less noble standard potential, but in the case of FeCo and FeNi alloys, the anomalous co-deposition occurs, thus the changes in the cathodic potential may lead to an unexpected chemical composition modification. Additionally, CV measurements performed for individual elements showed different behavior of Fe, Co, and Ni ions under the applied potential. Therefore, SEM and EDS measurements were performed to investigate the morphology and chemical composition of FeCo and FeNi NWs deposited at various potentials or from different electrodeposition media composition.

### 3.2. Morphology and Chemical Composition of Nanowires

**Figure 3:**
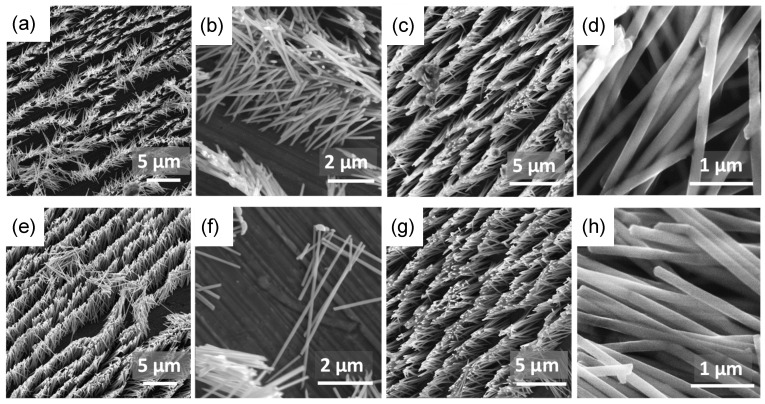
SEM images obtained at two different magnifications for (**a**–**d**) FeCo NWs deposited at (**a**,**b**) −1.0 V and (**c**,**d**) −2.0 V, and for (**e**–**h**) FeNi NWs deposited at (**e**,**f**) −1.0 V and (**g**,**h**) −1.6 V, as observed after membrane dissolution.

The morphology of NWs was studied after membrane dissolution by scanning electron microscopy. The micrographs show that there are no significant differences between FeCo and FeNi nanowires, regardless of the electrodeposition solution composition and the applied cathodic potential (Figure 3). In all samples, the nanowires create densely packed nanostructures, forming matrices of nanowires with uniform diameters and lengths. No effect of composition changes, deposition rate, and hydrogen evolution on the morphology was observed. All nanowires were smooth and continuous, without noticeable porosity.

**Figure 4:**
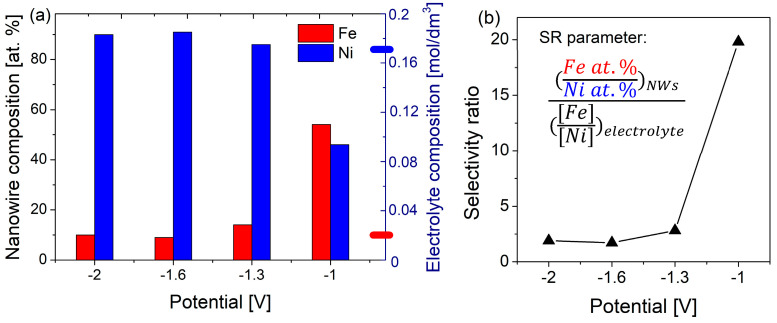
(**a**) Atomic composition of Fe and Ni in FeNi NWs deposited at different potentials, with the marked position of the molar content of Fe (red) and Ni (blue) in the electrodeposition solution on the right axis; (**b**) selectivity ratio of Fe/Ni, calculated for FeNi NWs. The particular elemental contents were given with an error of no more than ±1.

EDS studies, showing the relative atomic concentrations of each element, were carried out on FeCo and FeNi NWs released from the membranes by dissolution of the polycarbonate matrix. In the case of FeNi NWs, changes in electrodeposition potential resulted in various compositions of nanowires (Figure 4), in contrast to FeCo NWs for which only a few percent changes in content are noticeable at extreme values of the applied potentials. Such poor sensitivity of the chemical composition of FeCo NWs to the applied potential is consistent with the literature [59,60,61], and will require another strategy (i.e., different compositions of the electrodeposition medium) to try obtaining FeCo NWs with variable Fe:Co atomic ratios (see Table 2 and related text below) in view of varying their structural and magnetic properties.

The Fe and Ni concentrations in FeNi NWs deposited at different cathodic potentials are plotted in Figure 4. It can be observed that the Ni content increases with the cathodic potential, while the Fe concentration decreases, in agreement with previous observations [36,62]. The variation of the Ni content from 46 to 90 at% suggests that, depending on the Ni concentration, the different phases predicted based on the phase diagram may be stable. This will be discussed in the next section. The Fe and Ni contents in the electrodeposition medium do not reflect the chemical composition of FeNi NWs, with nanowires deposited at −1.0 V, indicating the occurrence of strong anomalous co-deposition. The Fe concentration in this sample, comparable with the Ni content, is much higher than the Fe^2+^ ion concentration in the synthesis solution (Ni/Fe molar ratio = 17), clearly showing the preferential deposition of iron. At more negative potentials, the Fe content decreases significantly, and at higher values, it maintains a constant low content. Although in this potential range the relative atomic concentrations in FeNi NWs approach the ion concentration ratio in the solution, this is still far from the elemental composition of the solution.

The high Fe content can be explained by the larger partial current originating from Fe^2+^ ions reduction at low potential due to anomalous co-deposition [63]. At more cathodic potentials, the maximum partial current corresponding to the reduction of Ni^2+^ ions is reached, which causes an increase and then saturation of the Ni content in FeNi NWs. The diffusion limit achieved at higher potentials for both Fe^2+^ and Ni^2+^ ions results in a potential-independent composition of nanowires [62].

Based on the obtained iron and nickel concentrations, selectivity ratio (SR) values were calculated to indicate the type of electrodeposition. The Fe^2+^/Ni^2+^ molar ratio in the electrodeposition medium used to obtain FeNi NWs was 0.01/0.17. The determined SR coefficients for FeNi NWs are presented in Figure 4b. An SR value close to 1 suggested co-deposition predicted by standard potentials and solution composition, while anomalous co-deposition occurred for a higher SR coefficient. The calculated SR parameters show a high value (close to 20) for electrodeposition performed at the less cathodic potential and closer to 1 (i.e., around 2) for more cathodic potentials. Hence, the observable anomalous co-deposition appears much more preferably at the lowest overpotential (i.e., −1.0 V), while applying higher cathodic voltages lets the electrodeposition process lose its anomalous character. These results confirm the influence of voltage on the anomalous co-deposition phenomenon. The FeNi alloy nanowires were found to be sensitive to the applied potential, which should affect their structural and magnetic properties.

The situation was different for FeCo NWs, as pointed out by chemical compositions of FeCo NWs electrodeposited from two distinct solution compositions, for which only few to ten percent increase in the Co content with rising potentials from −1.0 V to −2.0 V was observed (Table 2), contrary to the case of FeNi NWs for which the use of extreme potentials resulted in an almost 100% increase in the Ni content (Figure 4a). Barely higher variations can be observed in more dilute electrodeposition solutions (Table 2). The selectivity ratio parameters are equal to 1 or slightly higher, but none of these values indicate strong anomalous co-deposition. According to the phase diagram, for all Co contents ranging from 53 to 66 atomic percent, only the FeCo BCC B2 phase is expected [24].

The above results suggest that it is possible to obtain segmented nanowires with distinct regions, characterized by various chemical compositions, from a single electrolyte bath by applying different values of cathodic potentials in the case of FeNi NWs. In contrast, this may be limited for FeCo NWs, showing poor sensitivity of the chemical composition to the applied voltage and electrodeposition solution composition.

### 3.3. Phase Composition

**Figure 5:**
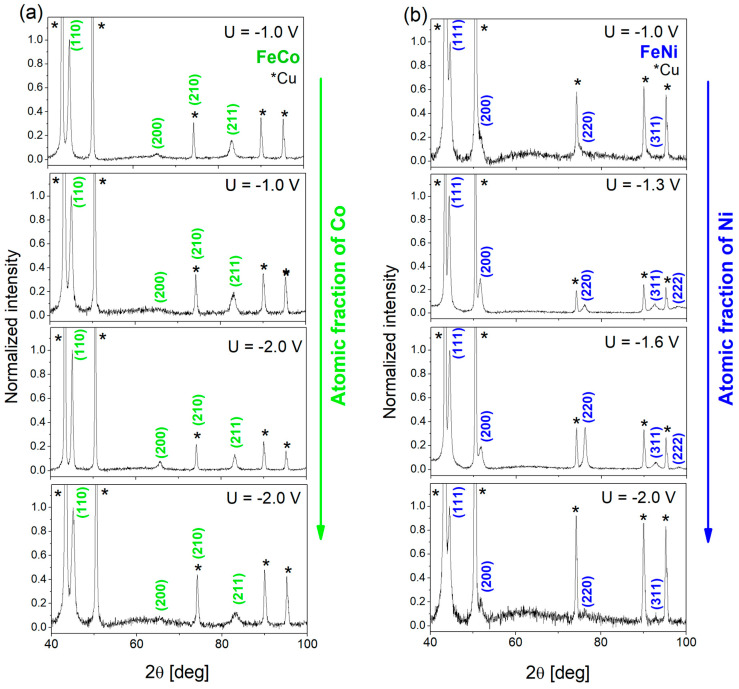
X-ray diffraction patterns measured for (**a**) FeCo and (**b**) FeNi NWs electrodeposited in PC membranes in different conditions (FeCo samples as in Table 2; FeNi samples as in Figure 3a). The indexed peaks correspond to the FeCo BCC and FeNi_3_ FCC phases (reference code: 03_065_6829 and 00-038-0419 NIST Database); the peaks marked with an asterisk come from the Cu layer.

The phase composition of the FeCo and FeNi alloy nanowires were examined by X-ray diffraction measurements. The measurements were carried out on samples embedded in the polycarbonate membrane and typical results are presented in Figure 5. The very intense peaks marked with asterisks come from the Cu underlayer. The peaks described by Miller indices were assigned to the FeCo BCC and FeNi_3_ FCC phases according to the reference data (03_065_6829 and 00-038-0419 NIST). The peak positions and their intensities are listed in Table 3 with the reference data. All nanowires exhibit a polycrystalline structure.

Independently on their composition, the analyzed FeCo NWs reveal the occurrence of the B2 phase. It is an ordered BCC structure with Fe (Co) atoms located at the corners (center) of the unit cells. The most intense diffraction peaks for FeCo alloy come from the (110) planes and appear at an angle of approximately 2θ = 44.9°. Two other peaks with lower intensities are assigned to the reflections from the planes (200) and (211). Relatively small changes in Co concentration do not influence the peak positions, which indicates no changes in the lattice parameters. Because of the superposition of the FeCo (210) and Cu (220) signals, the detailed analysis of their relative intensities may be biased, but comparable intensities of the Cu peaks at 2θ = 74.3° and 2θ = 90.2° suggest minimal FeCo contribution to the overlapping peak, indicating a rather isotropic growth of the FeCo NWs.

In the case of FeNi NWs, according to the phase diagram and the Ni content (46–90 atomic %), one can expect the appearance of FeNi (at the lowest Ni concentration, U = −1.0 V) and FeNi_3_ phase (at higher Ni content, U = −1.3 ÷ −2 V). The peak positions were assigned to the reference data of the FeNi_3_ phase with the most intensive peak at an angle of approx. 2θ = 44.4° coming from (111) planes. The slight deviation from the peak position showed no correlation with changes in chemical compositions. The relative peak intensities indicate the preferred growth direction normal to the (111) planes ([111] direction), with one exception for the sample deposited at −1.6 V, which also shows slight texture along the [110] direction (normal to the (220) plane). The high peak intensities found in the samples deposited at −1.3 V and −1.6 V may suggest that intermediate voltages create the most favorable conditions for the growth of crystalline FeNi NWs. The analysis did not reveal the peaks typical of the FeNi phase.

### 3.4. Magnetic Studies

**Figure 6:**
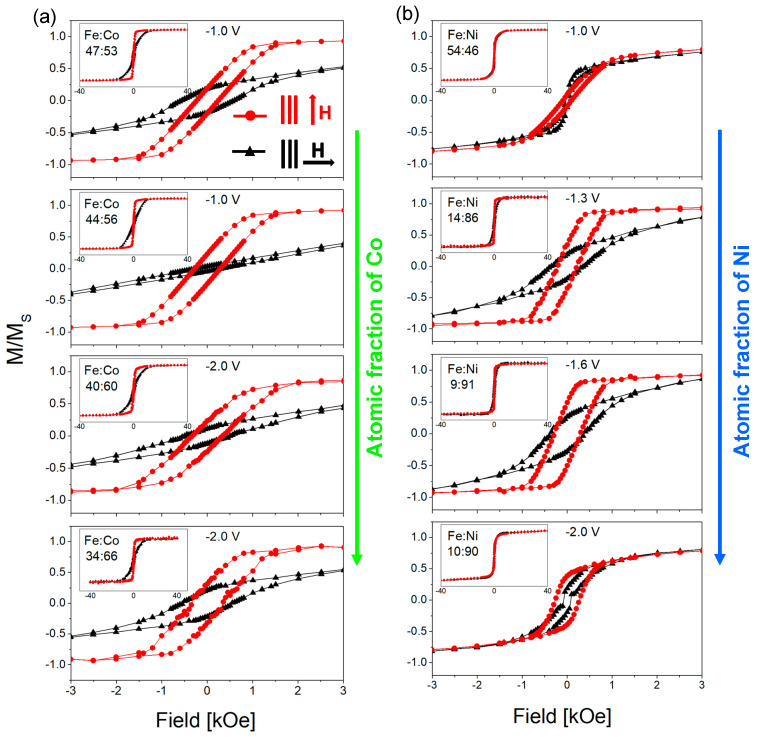
Hysteresis loops measured at room temperature with a magnetic field applied in the membrane plane (H ꓕ NWs—black triangles) and out of it (H II NWs—red circles) for FeCo (**a**) and FeNi (**b**) nanowires (Φ = 100 nm) of different compositions (FeCo as in Table 2 and FeNi as in Figure 3a). The magnetization (M) value was normalized to the saturation magnetization (M_s_).

FeCo and FeNi NWs were also studied magnetically on the basis of SQUID measurements. Figure 6 presents the hysteresis loops of FeCo and FeNi NWs of different chemical compositions (the same samples as in Figure 5). Magnetic measurements were performed at room temperature with a magnetic field applied in the membrane plane and along the normal to the membrane plane.

**Figure 7:**
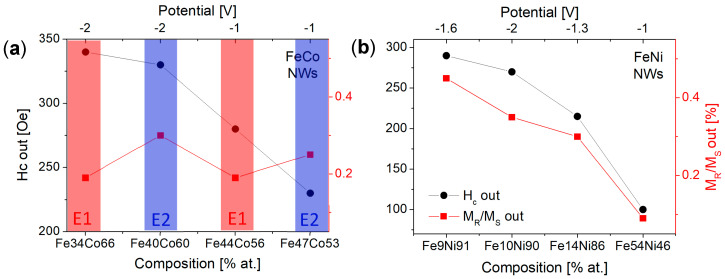
Out-of-plane coercivity and squareness shown as a function of sample composition (and cathodic voltage) for (**a**) FeCo and (**b**) FeNi nanowires.

From the hysteresis loops in Figure 6a, it can be seen that all FeCo NWs are characterized by magnetic anisotropy with the easy axis along or near the nanowire axis. The lack of a preferred growth direction excludes the magnetocrystalline anisotropy from the factors contributing to the magnetic behavior of FeCo NWs. Thus, the magnetic anisotropy may result from shape anisotropy and dipole interactions. The loops measured with a magnetic field applied out of the membrane plane deviate from the rectangular shape. This shape may be associated with the deviation of the channels from the normal to the membrane surface or relatively strong dipolar interactions. The in-plane curves, except for one sample, are open, which indicates non-coherent rotation related to the domain wall motion. Increasing the Co content causes an increase in the out-of-plane coercivity and non-monotonic changes in the squareness (Figure 7a). It can be noticed that the samples deposited at a more negative voltages have greater coercivity compared to samples deposited from the same electrolyte at low potential. This can be associated with a higher deposition rate, a more porous structure, and a lower filling degree (lower charge Figure 2a). A more porous structure with more defects can create the pinning sites for the domain movements, while a lower filling degree reduces dipole interactions. These changes may be responsible for the observed increase in the coercivity values. The analysis of the applied potential allows us to explain the non-monotonic squareness behavior. As can be seen, the samples prepared at different voltages but from the same electrolyte show almost identical squareness values, which further confirms the poor sensitivity of the FeCo NWs to the applied cathodic potentials.

Figure 6b shows the hysteresis loops measured for FeNi NWs. It is clearly visible that there is no direct correlation between the shape of the hysteresis loop and the Ni content in the nanowires. The main parameter determining the magnetic behavior of FeNi NWs seems to be the applied voltage. Samples deposited at extreme voltages (with Ni contents of 90% and 46%) are rather magnetically isotropic, while samples deposited at intermediate voltages (containing 91% and 86% of Ni) exhibit magnetic anisotropy with an easy axis along the nanowire axis. As shown, the samples deposited at intermediate potentials had a better crystalline structure, in contrast to samples deposited at extreme potentials, which were characterized by low peak intensities in the diffractograms. Thus, structural differences, especially poor crystalline structure, may be responsible for the isotropic behavior of nanowires. The preferred growth direction along the magnetic easy axis ([111] direction) did not enhance magnetic anisotropy, suggesting its marginal contribution to the effective anisotropy. Although the coercivity and squareness increase with increasing Ni content, only the samples showing magnetic anisotropy are interesting from the application point of view (Figure 7b).

The changes in the chemical composition of nanowires due to the modification of the electrodeposition solution composition (metal ions concentration) turned out to be significant for FeNi NWs, but in this case, the extreme potential values applied led to a magnetic anisotropy loss. In the case of FeCo NWs, the changes in the potential did not indicate a significant modification of the chemical composition, however, the increasing Co content, achieved by changing the concentration of Fe and Co in the synthesis solution, resulted in an increase in the coercivity of FeCo. The changes in the chemical composition and associated various magnetic parameters, especially coercivity, may lead to the creation of a boundary between soft and hard materials, which could become a desirable center for pinning the domain wall. An interesting observation revealing the different sensitivity of FeCo and FeNi alloys to the applied potential and the appearance of the anomalous co-deposition in FeNi NWs deposited at low potential demonstrates the different impacts of Co and Ni on the behavior of the alloy.

The idea of producing 3D magnetic memory in the form of segmented nanowires prepared by applying various cathodic potentials turned out to be a non-trivial task because of the poor sensitivity of the chemical composition of the FeCo system and the loss of the perpendicular anisotropy in the case of FeNi NWs deposited at applied extreme potentials. This might be taken up using three-element FeNiCo nanowires.

## 4. Conclusions

FeCo and FeNi NWs deposited at different cathodic potentials and/or various electrodeposition solution composition behaved in distinct ways. While FeNi NWs showed significant changes in their chemical composition upon applied voltage, FeCo NWs demonstrated only a few percent modification of the element contents. Moreover, strong anomalous co-deposition was observed in FeNi systems at the less cathodic potential, while FeCo NWs reflected the solution composition quite well. Current vs. potential curves measured for the FeCo system seemed to indicate a slightly easier reduction compared to synthesis solutions containing separate Fe^2+^ and Co^2+^ ions, with similar reduction efficiency. In contrast, CV analysis for the FeNi system pointed out the reduction at less cathodic potentials and much higher current at the main wave, suggesting more efficient reduction during alloy formation. As expected, these binary alloys crystallized in different structures (FCC and BCC), but FeCo NWs showed rather isotropic growth, while FeNi NWs exhibited a clear texture along the easy magnetic axis. The coercivity of both systems increased with decreasing Fe content, with the same trend for the squareness of the FeNi system and squareness dependent only on the electrolyte in FeCo NWs. Thus, FeCo and FeNi NWs showed various behaviors under the influence of the cathodic potential, but neither of them met the requirements for racetrack 3D memory.

## Figures and Tables

**Table 1:** Composition of the various electrodeposition media used in this work.

Solution Number	Composition of the Medium					pH
	FeSO_4_	CoSO_4_	NiSO_4_	H_3_BO_3_	C_6_H_8_O_6_	
	[g/L]/[mol/dm^3^]					
E1	16.68/0.06	33.73/0.12	-	24.72/0.4	0.5/0.003	2.8
E2	2.78/0.01	5.62/0.02	-			
E3	2.78/0.01	-	44.71/0.17			3.2

**Table 2:** Chemical concentration/content of Fe and Co species in both the synthesis solutions and the nanowires electrodeposited at −1.0 or −2.0 V.

Potential [V]	Solution Composition [mol/dm^3^]		Molar Ratio in Solution Fe:Co	Nanowire Composition [%]		Atomic Ratio in Nanowires Fe:Co	SR Parameter
	FeSO_4_	CoSO_4_		Fe	Co		
−1.0	0.06	0.12	0.5	47	53	0.9	1.8
−2.0				44	56	0.8	1.6
−1.0	0.01	0.02	0.5	40	60	0.7	1.4
−2.0				34	66	0.5	1.0

**Table 3:** XRD measurement results for FeCo and FeNi nanowires compared to reference * data (03_065_6829 and 00-038-0419 NIST Database).

**FeCo**					
**hkl**	Reference *	**Fe47Co53** **−1.0 V**	**Fe44Co56** **−1.0 V**	**Fe40Co60** **−2.0 V**	**Fe34Co66** **−2.0 V**
2θ [deg]					
110	44.94	45.00	45.06	45.08	44.96
200	65.43	65.54	-	65.58	-
210	74.35	74.23	74.20	74.23	74.22
211	82.90	83.07	83.14	83.18	83.01
220	99.7	-	-	-	-
Relative Intensity [%]					
110	100	100	100	100	100
200	11.3	6	-	8	-
210	0.1	31	35	22	44
211	17.0	16	20	14	13
220	4.4	-	-	-	-
**FeNi**					
**hkl**	Reference *	**Fe54Ni56** **−1.0 V**	**Fe14Ni86** **−1.3 V**	**Fe9Ni91** **−1.6 V**	**Fe10Ni90** **−2.0 V**
2θ [deg]					
111	44.28	44.42	44.31	44.43	44.44
200	51.53	51.52	51.59	51.72	51.67
220	75.87	-	75.9	76.14	-
311	92.21	-	92.53	92.68	92.85
222	97.70	-	97.96	98.02	-
Relative Intensity [%]					
111	100	100	100	100	100
200	60	20	29	20	24
220	30	-	8	35	-
311	40	-	8	7	11
222	10	-	6	3	-

The crystallite sizes calculated based on the Scherrer equation, with an error of ±3 nm due to low peak intensities and their proximity to the Cu peaks, were estimated to be below 20 nm for both alloys.

## Data Availability

The original data presented in the study are openly available in The Repository of the Institute of Nuclear Physics, Polish Academy of Sciences at DOI: https://doi.org/10.48733/no3.25.018.
